# Retiring from Business

**Published:** 1880-11

**Authors:** 


### RETIRING FROM BUSINESS.

Thousands of men in New York, Philadelphia, Baltimore, Boston, and other business centres, who commenced operations from a quarter to a half century ago, are still on 'Change. A part of them have done well, are doing well, and could continue to do well longer.

Meanwhile, the young men are asking with impatience, when will these old fellows die off and give us a chance? Young America is quite sure it would be contented with half, or a quarter of what the old men have gathered; and would retire with an excellent grace, and give place and elbow-room to another generation.

To the young men, we would say: not quite so fast; the fathers have showed this long time a will and an energy, that cannot easily be hurried, and you may as well be a little patient, since they will either await a higher summons than yours, or will withdraw from the marts of commerce very much at their own discretion, as you will wish to do by and by, if you should first be as successful as they have been.

For older business men, successful, prospered, rich—which in the parlance of the day, means far more wealth than their own or their childrens' best good requires—the question of retirement is a hard one. Their associations are all mercantile. Their habits are all business habits. Money-making is their life, comfort, health, everything. To take them from the theatre of their successes, might prove too much like drawing the finny tribe from the water, and the feathered races from the air, and dooming each to the element designed for the other. Not a few retiring men have been very happy while building themselves a palace, because thus far they were trading, manufacturing, using every day their natural and acquired skill in bargaining, in daily association with business men; but have been miserable from that day forth. They have found great pleasure in the laying out and construction of a princely home to die in; but were unreconciled to remaining and dying amid the awful stillness that reigned, when the noise of the saw and hammer had ceased. The singing of birds had less music for their ears, than the rumbling of wheels on cobble-stones; the green earth and blue sky pleased them less than brick and mortar; and, the first you know, they have sold an earthly paradise for half its cost, and are back to their old haunts, as much in the way of their juniors as ever, but some tens of thousands less rich for the operation. Such examples are a damper.

Others again seem to enjoy rural life with a zest known only to those who have been long deprived of it. "God made the country, man the city;" and they feel that God's works are a thousand times the loveliest and best. A quiet old age and a graceful departure are the result of their retirement. Here then are arguments pro and con, the experience of one class balancing that of the other, and leaving the question undecided.

In favor of perseverance in business to the end of life, it may be said: how easy for the elderly man to make money; he has the use of all the ropes, capital, practical skill, knowledge of men, extensive acquaintance, old customers. He can make more in one year now, than he could in three, at his first setting out. But in reply to this, it is unquestionably true that old men sometimes lose by adhering too long to the walks of business. Times change; modes of doing business, to which they were unused in their better days, are introduced; the young men are quicker to learn new things; and the old man finds himself left behind in the race. Here then is another balance. If the elderly business man has advantages, he also labors under disadvantages, as compared with the young.

May it be urged that the successful business man should retire partly in favor of his juniors? Some of them have served him, have contributed, it may be supposed, to his success, and are deserving of his esteem and warm friendship. If he has paid them well, allowing something like a fair division of the profits, it would be difficult to fasten upon him an obligation to yield his place and advantages to them; but it would certainly be the mark of a manly, generous mind, to be influenced by such a consideration—to say, in retiring, that he has enough, and rejoices to leave the young men, who have served him, in a better position than his was, at their stage of life.

But the question of retiring or holding on, will depend very much upon the man himself, what he was in the outset of a business career, what business has made him, and what he purposes to become.

If he is physically one of those nervous, active, stirring men, who never can be comfortable except when uncomfortably hard at work, never easy except when driving ahead, one who *must* work as long as he lives, and *must* die when he can be active no longer, such as are some of the very best of men, it is better that he should enlarge rather than abandon his business. Such men never can rest; but they generally wear like steel; and the more you give them to do, the better they wear. They are uncomfortable to themselves and every body else, unless they have a little more to do than they can possibly do; and they can, in old age, do that which they have been accustomed to, better than something new.

Again, if you are selfish by nature, and made doubly so by the competitions of trade, close-fisted, rapacious, hoarding, or, what is hardly better, rolling over and rolling up for yourself alone; extending your sympathies hardly beyond the family circle, and not at all beyond the cousin-hood and nephew-dom; recognizing no obligations to men outside of your own little circle; anxious to pay your note, because it is for your interest to pay it, but willing your washer woman should call for her pay a third and a fifth time, since she cannot hurt you; in short, if nature and trade have made you a hunks and a miser, we advise you to stick to your business; not a fig need you care for the young men who want your place; and as for the brotherhood of the human family, it will have to take care of itself, as best can. Go on, accumulate and hold fast, and God will see that your wealth falls into better hands, when you can hold it no longer.

But if, on the other hand, you have cultivated a generous nature, learned to feel for others' woes, to comfort the desponding, to reclaim the erring, to feel towards every one whom you have it in your power to aid as towards a brother, and if you really believe that you can see your property stationary or decreasing, without mourning over it, the hungry fed from it, the naked clothed, the ignorant taught, want and suffering relieved, then we very much suspect that better and happier work awaits you, than this dabbling, all the way down to the grave, after more wealth. Do not think to be happy by inaction. Nothing is more absurd, than to expect that you can pass all at once from a state of extreme activity to one of indolence, and be contented. Every law of mind and body forbids such a hope. Neither can you expect to be contented with any splendor which your wealth might procure, nor with mere trifling. Gunning, fishing, a walk, a drive, gossip by the way, an innocent game, a snooze after exercise, are all very well in their place. But a man of your high-born, noble sentiments, needs to think that he has done something useful that day, before he can enjoy any one of these, and that these are but his recreations, not the things for which he lives. If you withdraw from business, continue to have about as many cares as before, and of a kind that you can feel are quite as important. Not he who has a thousand cares, but he who has none, is the really wretched man. Make yourself liable to be called upon, to go, now here and now there, on some good errand. Of all things, contrive to get hustled and rubbed a good deal. A man of your habits, cheated by turns all his life, and having saved himself at other turns only by keeping a sharp look out, needs a good deal of shaking up and rubbing to keep him bright and free from rust in old age. Make up your mind to be active and useful, enterprising to do good.

In your former employment it was pleasant to make good bargains, if you could make them and wrong no one. Now you can make better bargains. Near you will be a poor widow, with a long row of children. She is willing to wash, iron, sew, anything to sustain and train up those children in the way they should go. God has promised that the widow and fatherless shall not want. He will not send Gabriel to carry them bread, He will expect you to fulfill his promise. Sometime, when there has been sickness in the widow's cottage, or work enough has not offered, she will fail by a few shillings of bringing the week out, and she will know where to go for the balance—will go to you, or, more probably, to your wife—for you will give her a chance at some of the good bargains—and will relate her efforts and the deficiency, and ask you to loan her six shillings, and you will not *loan* her six shillings, but will *give* her a dollar, and wish she had asked for more. She will value the dollar as much as you would a hundred, and so you have a hundred for one; and more, every one of her four children will be as grateful for that dollar as you would be for a hundred; and now you have five hundred for one; and remember that "He that giveth to the poor lendeth to the Lord," and the Lord will certainly allow good interest and pay up. So then if you retire from the marts of business, you can make better bargains than ever. A smart, enterprising boy will need another quarter's schooling before he goes to a trade, but there is no one to pay the five dollars tuition. You will pay it, and he will love you, and talk about you, as long as you live, and after you are dead.

